# Empowering education for ophthalmologists

**Published:** 2017

**Authors:** T. Horia Stanca

**Affiliations:** * RSO Member of the Board AAO’s Regional Advisors Committee Member

Romanian Society of Ophthalmology (RSO) and its Partner, the American Academy of Ophthalmology (AAO) are empowering education for Romanian Ophthalmologists by online clinical education resources.

There have already been 2 years since RSO signed a partnership with AAO in order to provide access to ONE Network for every Romanian ophthalmologist, this opportunity being welcomed by our members, and being more and more used.

Ophthalmic News and Education (ONE) Network is a Global Education Platform for Ophthalmologists launched in November 2007, which nowadays offers access to more than 400 courses and interactive cases, more than 15000 pages of ophthalmic content, more than 3600 clinical images, more than 1800 instructional videos and podcasts, 10 leading scientific journals, these numbers increasing constantly. ONE Network is a great resource for innovative education addressed to trainees offering 24 courses in all subspecialties, more than 100 videos and AAO lectures and more than 1000 self-assessment questions. This online resource for clinical information and instruction also includes up-to-date clinical guidelines translated into 15 languages, including Romanian.

AAO collaborates with the Ophthalmology Societies Worldwide and ONE Network, through the Global Alliances, which is an outstanding project that had a significant impact on both clinical education and the delivery of eye care. 

Last year, during the 2016 AAO Meeting in Chicago, Richard L. Abbott, MD, Secretary for Global Alliances and Past President of the American Academy of Ophthalmology, has unveiled the “Global Directory of Training Opportunities”, a service that will help the ophthalmologists worldwide identify the training opportunities (observership, fellowship or preceptorship) outside their country and expand their knowledge and instruction in the United States and internationally. Working with the AAO’s Regional Advisors Committee, the Global Alliances has succeeded in just one year to plan and launch a Global Observership Listing Service and this tremendous option can be accessed by every Romanian ophthalmologist through ONE Network platform (aao.org/training-opportunities).

The Romanian Society of Ophthalmology is proud to be one of the 70 national Societies that can offer ONE Network as a benefit for their members to stay updated and get the most comprehensive schooling.

In addition, dear Romanian Ophthalmologists, feel free to expand your knowledge and identify the best educational opportunities by using ONE Network platform!

**Stanca T. Horia MD, PhD** **RSO Member of the Board** **AAO’s Regional Advisors Committee Member**

